# Dermatology teaching for undergraduate medical students in clinical routine – a structured four-week curriculum

**DOI:** 10.1186/s12909-023-04921-x

**Published:** 2024-02-06

**Authors:** Tobias Kliesener, Madeleine Jandek, Alexander Navarini, Oliver Brandt, Simon Müller

**Affiliations:** Dermatologische Klinik, Universitätsspital Basel, Basel, Switzerland

**Keywords:** Undergraduate medical student, Medical education, Dermatology, Teaching, Curriculum, Generation Y, Generation Z

## Abstract

**Background:**

Dermatology teaching is fundamental for the promotion of young colleagues in our specialty. However, traditional teaching methods are being scrutinized by students of the ‘Generation Y and Z’, which can pose new challenges for teaching institutions. We therefore aimed to assess the motivational impact and reception of a newly created four-week curriculum containing modernized teaching methods integrated into clinical routine.

**Methods:**

In this single-center study, 67 medical students completed this curriculum composed of weekly learning objectives including knowledge of morphological terms, 10 common dermatoses, communication and presentation skills. The participants provided information on their level of interest in dermatology each week as well as positive and negative aspects of the curriculum.

**Results:**

During the curriculum a significant median increase in interest in dermatology was reported with no differences between the genders. Low initial interest could be improved, high initial interest maintained. Participants with an interest in scientific work (20.9%) were more motivated during the curriculum.

The variety, quality of teaching and structure were the main aspects rated positively. Suggestions for improvement included the need for more teaching by senior doctors, transfer of responsibility, and a working environment updated to the latest technology standards.

**Conclusion:**

The presented curriculum was well received by the participants and allowed to better define learning preferences of new generations which can be helpful to modernize traditional teaching methods. Interest in scientific work could be a factor to identify students with a particularly strong interest in dermatology.

**Supplementary Information:**

The online version contains supplementary material available at 10.1186/s12909-023-04921-x.

## Background

Dermatology training of medical students is a fundamental requirement to ensure good basic knowledge and clinical skills in future physicians [1] and, perhaps even more importantly, to ignite the spark of passion for this specialty [2, 3]. This becomes particularly crucial as cutaneous diseases are a primary reason for health consultation [4], making non-specialist often the gatekeepers in the initial assessment of skin conditions [5, 6]. However, there might be a substantial number of them receiving inadequate dermatology training during their undergraduate studies [5, 7]. Addressing these educational challenges have occupied dermatology teachers since decades [8]. Furthermore, the evolving wishes and expectations of new generations entering the workforce make the adaption and development of teaching methods in dermatology necessary. Today’s student population is primarily composed of the so-called Generation Y (1980–1994) and Generation Z (1995–2015) [9]. These generations have grown up in the digital age, in which learning is seen as an active experience with a high level of participation [10]. Furthermore, they prioritize a work environment based on the latest technologies [10], flexible working conditions and productive exchange with their employers, including regular feedback and recognition [11, 12]. These preferences present challenges to traditional teaching models and worsen existing ones, such as the shortcoming to provide adequate training to fulfill the objectives of undergraduate dermatology education using solely lecture-based approaches [5, 13]. Additional challenges include the absence of standardized tutor training and leadership development [14], limited number of tutors [15] and some teachers’ resistance for change [16]. A scoping review by Bernges et al. [17], summarized the wide range of studies examining the impact of different dermatology teaching approaches for medical students. It showed that activating, diversified, and knowledge-testing teaching approaches delivered by didactically trained tutors, increase the impact of the learning success [17]. Previous studies have primarily focused on subjective (e.g. self-assessments [18]) and objective (e.g. knowledge tests [19]) teaching effects, whereas little attention has been paid to students’ perceptions and their suggestions for improving the curriculum [20]. It appears that there is no evidence-based concept on how to adapt students’ education accordingly. Therefore, critical review and evaluation of new curricula is essential for improving future generations’ education [21].

Previously, our clinic lacked a standardized curriculum for student internships, leading to inconsistent personal benefit and varying levels of satisfaction among students. Dedicated students couldn’t fully benefit from their internship, while less motivated ones weren’t adequately challenged. We therefore aimed to introduce and assess a four-week curriculum consisting of modular teaching content, which incorporates recommended, modernized teaching methods [17]. The curriculum aimed to foster external motivation through weekly learning objectives, knowledge testing, and the obligation for an oral case presentation. At the same time, the intrinsic motivation of students should be promoted through a positive working environment and extensive integration into everyday working life. The students had the opportunity to engage in additional challenges (e.g. scientific work) and to recognize the relevance of dermatology through the variety of learning materials (e.g. flashcards, websites) or the chance to care for patients independently but under supervision.

The primary objective of this study was to determine the impact of the curriculum on students` interest in dermatology. Therefore, we surveyed undergraduate medical students about their interest in dermatology over time, assuming that a well-structured curriculum would improve it. The secondary objectives were a.) to examine, possible differences in the perception of the curriculum based on gender and interest in scientific work and b.) to improve our understanding of how to optimize dermatological education for future generations. To this end, we determined and evaluated the positive aspects and suggestions for improvement regarding the curriculum and present the most common take-home messages mentioned by the students.

## Methods

### Participants

The analyzed data was collected between June 2020 and April 2023 at the Department of Dermatology of the University Hospital Basel, a tertiary referral center offering the entire spectrum of diseases of a central European dermatology and allergology university clinic. The clinic provides an outpatient clinic each for dermatology and allergology, a 12 beds ward, a dermatosurgery unit and a clinic for aesthetic dermatology. In 2022, a total of 450 patients were treated in the ward, with an average hospital stay of 5.4 days (sd = 0.6) and 72,260 outpatient consultations were held.

The participants in this study were medical students attending their internship year in our department during their fifth year of medical school. A maximum of 5 students were enrolled in this four-week curriculum at the same time to allow all rotations to different stations and positions. In none of the participants’ country of study, participation in a dermatology internship is mandatory, which means all participated voluntarily based on their individual interest in dermatology. All undergraduate students attending our clinic for at least 4 weeks between June 2020 and April 2023 were eligible to participate in the curriculum. In total 71 undergraduate medical students participated. Data of students were excluded in case of incomplete participation (*n* = 1) or if their questionnaire was not completed (*n* = 3). The selection of participants through their dermatology internship in our department can be categorized as purposive, non-probability sampling. The study was conducted in accordance with the Declaration of Helsinki and the Ethics Committee of the University of Basel. The study and the curriculum received approval from the University Hospital Basel and the Head of the Dermatology Department. Informed written consent was given by all participants. The data in this study was anonymized before its use. Participants received no participation fee.

### Curriculum

**Fig. 1:**
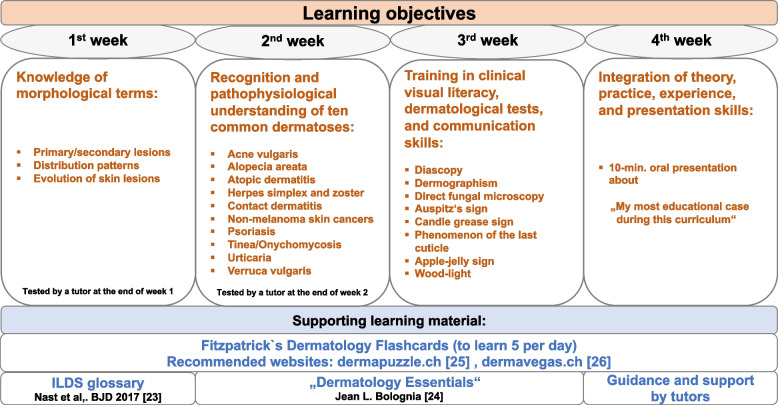
Overview of the structured four-week curriculum in dermatology. ILDS: International League of Dermatological Societies

The curriculum, integrated into clinical routine, consisted of a four-week period that sets new learning objectives each week as shown in Fig. 1. Its content was structured as follows:

In week 1: The learning objectives were the recognition and correct use of morphological terms such as primary/secondary lesions, their distribution patterns and evolution (e.g., in German called “Effloreszenzenlehre”). Week 2 focused on the recognition and pathophysiological understanding of 10 common dermatoses detailed in Fig. 1 with the aim to apply and practice the learning objectives of the first week alongside. This basic knowledge was complemented in week 3 by training of the visual literacy and bedside repetition of the learned topics and also by newly learned clinical skills such as dermographism, direct fungal microscopy (more details in Fig. 1) and also by practicing communication skills. The synthesis of all the learning objectives culminated in an oral presentation about ‘my most educational patient case’ in the final, fourth week. The learning objectives of week 1 and 2 were tested by an assigned tutor (usually a resident of the department) during predefined time slots.

The students have spent 2 weeks each at the inpatient ward and the dermatology outpatient clinic. In addition, trial days in the clinics of surgery, allergology, and aesthetic dermatology were inserted. Supporting learning material, such as handouts [22], recommended books [23], or websites covering the learning content, were provided to the students. Additionally, each student received the Fitzpatrick’s Dermatology Flashcards, with the instruction to learn a minimum of 5 flashcards per day in self-study. For the repetition of teaching content, further in-depth study and especially training in clinical visual literacy, the websites dermapuzzle.ch [24] and dermavegas.ch [25] were recommended for self-study as well.

The curriculum was created by SM who is responsible for the students’ internship since 2019. His draft was critically reviewed by the head of department (AN) and the secretarial staff responsible for the organization of the curriculum and students’ employment contracts. Following minor adjustments, the curriculum was tested in a pilot-phase of 1 month duration, thereafter due to the positive feedback both from the students’ and clinical staff, adopted without additional changes. Since training requirements vary widely internationally, the external framework with a total duration of 4 weeks and weekly learning objectives may not be directly adopted in many countries. However, it can be modified, retaining the core topics of conservation, namely “Effloreszenzenlehre”, common dermatoses, and diagnostical skills, as well as the consecutive character of the curriculum.

### Data collection

The degree of interest in dermatology was assessed on day 1, 7, 14, 21 and 28. Responses were provided on a numerical 10-point scale, with higher values indicating greater interest (minimum = 0; maximum = 10). Students’ perception including, a.) the positive aspects, b.) suggestions to improve the curriculum and c.) take-home messages were assessed using three open-ended questions with free text options. All responses in these domains were used for qualitative data analysis only. This form of data collection did not allow to statistically test any reliability or validity. Additionally, at the beginning of the curriculum, participants were asked about their interest in participating in a small scientific project. In case of a positive response, they were asked to contact a tutor/ senior physician in this regard within 3 days.

### Statistical analysis

The analyses were conducted using SPSS (IBM Statistics, Version 29) following consultation with the statistical support provided by the Clinical Trials Unit of the University of Basel. Normal distribution of values was tested using the Shapiro-Wilk test. Non-normally distributed data were presented using the median and interquartile range (IQR). The Mann-Whitney test was used to examine differences between two independent samples with non-normally distributed data. For multiple independent samples with non-normally distributed data, the Friedman test was applied, followed by a Bonferroni-corrected post-hoc analysis to show significant differences between each pair of the independent samples.

We employed a deductive-inductive mixed category system for the qualitative content analysis of the free text responses regarding the positive aspects, suggestions for improvement, and take-home messages. Initially, we established a general category list for evaluation and expanded it during data analysis with categories that emerged from the participants’ responses. For the positive aspects and suggestions for improvement, we formulated the general categories of ‘didactics/tutors’, ‘variety’, ‘recall learning objectives’, ‘participation/activation’, and ‘practical exercises’, which we derived directly from the teaching approaches promoted by Bernges et al. [17]. The category ‘didactics/tutors’ describes the support and training students receive, as well as the educational implementation of these methods through their tutors. The category ‘variety’ encompasses all points related to the diversity and variance of the curriculum, including rotation opportunities, teaching materials, and varied work. The category ‘recall learning objectives’ describes the verification of the acquired knowledge through the retrieval of learning objectives by the tutors. In the category ‘participation/activation’ perspectives on independent, activating, and inclusive learning approaches were presented, while the category ‘practical exercises’ gathers statements regarding hands-on activities. After reviewing the data, we extended the positive aspects and suggestions for improvement to include the categories of ‘time management’ and ‘structure/organization’. Additionally, we added ‘personal development’ as a positive aspect and ‘no improvement necessary’ as a category for the suggestions for improvement. We used the general categories of ‘skills in patient treatment’ ‘factual knowledge,’ ‘ethical values,’ ‘communication skills,’ ‘information management,’ and ‘coping skills’ for the analysis of the take-home messages. These categories have already been established for evaluating higher education [26]. The ‘skills in patient treatment’ category encompasses crucial skills gained for effective therapy like anamnesis, diagnostic procedures (biopsies, cryotherapy), and considering differential diagnoses, or independent and interdisciplinary work. ‘Factual knowledge’ included disease-specific knowledge, which in some cases also exceeded the necessary basic knowledge. ‘Communication skills’ included dealing with colleagues, patients, and feedback, while ‘information management’ referred to the research and application of current literature. When participants gained skills in coping with stress, this was attributed to ‘coping skills’, while personal growth or better identification with the profession was attributed to ‘ethical values’.

## Results

A total of 71 participants completed the four-week curriculum. Of these, 67 participants filled out the data collection entirely, the remaining (*n* = 4) were excluded due to incomplete data collection. The 67 participants were born between 1987 and 1999 (mean 1995), 48 (71.6%) were females. The majority of participants were from Switzerland (*n* = 52; 77.6%), the others from Germany (*n* = 11; 16.4%), Austria (*n* = 1; 1.5%), Italy (*n* = 1; 1.5%), Poland (*n* = 1; 1.5%), Czech Republic (*n* = 1; 1.5%), Croatia (*n* = 1; 1.5%) and two participants did not provide their country of origin (*n* = 2; 3.0%). For qualitative content analysis, all individual answers given by the participants in the data collection were taken into account, whereby ‘n’ corresponds to the number of responses given by the participants to the individual questions and is distributed among the three domains investigated as follows: a.) positive aspects (*n* = 199), b.) suggestions for improvement (*n* = 95), and c.) take-home messages (*n* = 255).

### Interest in dermatology

**Fig. 2:**
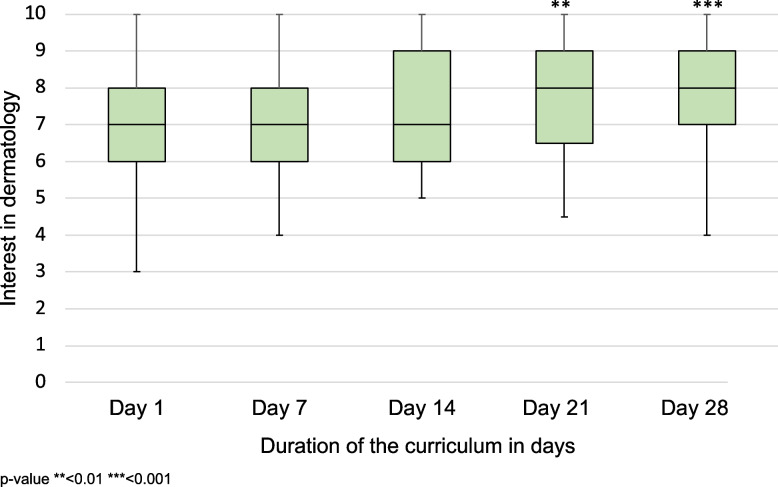
Comparison of the participants’ median interest in dermatology at different points in time compared to day 1 (*n* = 67)

Figure 2 displays how participants’ interest in dermatology changed at day 1, 7, 14, 21, 28 of the curriculum. A significant median increase in interest was observed on day 21 (7.0 vs. 8.0; *p* = 0.001) and day 28 in comparison to day 1 (7.0 vs. 8.0; *p* < 0.001). A comparison of the participants’ median interest in dermatology at different points in time can be observed in an additional Table A1 in more detail (see Additional file 1).

**Fig. 3:**
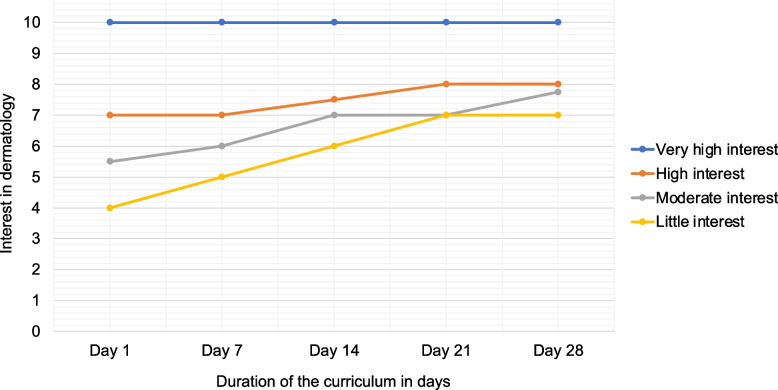
Progression of participants’ median interest in dermatology at different points in time (*n* = 67), grouped according to their level of interest at baseline

Figure 3 shows the participants’ median interest progression in dermatology at different points in time. To get a better idea of the individual change of interest during the curriculum, we stratified the participants into five groups based on their initial rating of interest on a scale from 0 to 10 NRS. No participants could be assigned to the lowest group ‘No Interest’ (according to 0–2 NRS). Participants were distributed as follows: ‘Little interest’ (according to 3–4 NRS; *n* = 6; 9.0%), ‘Moderate interest’ (5–6 NRS; *n* = 20; 29.9%), ‘High interest’ (7–8 NRS; *n* = 32; 47.8%) and ‘Very high interest’ (9–10 NRS; *n* = 9; 13.4%).

As shown in Table 1, participants were categorized into two groups based on their interest in undertaking a scientific work, which was extracurricular. The majority (*n* = 53; 79.1%) showed no interest in a scientific work. Their interest in dermatology was then evaluated on day 1 and day 28. Participants with interest in such a scientific work exhibited a significantly stronger interest in dermatology on day 1 of the curriculum (*p* = 0.02) which increased further until the end of the curriculum on day 28 (*p* < 0.001).

**Table 1:** Comparison of participants’ interest in dermatology based on their interest in scientific work on day 1 and day 28 of the curriculum (*n* = 67)

	Total	No interest in scientific work	Interest in scientific work	*p*- value
**Participants** n (%)	67 (100)	53 (79.1)	14 (20.9)	
**Median Interest (day 1)**	7.0	7.0	8.0	**0.02**
0.25 and 0.75 quantiles	{6.0–8.0}	{5.0–8.0}	{6.0–10.0}	
IQR	2	3	4	
**Median Interest (day 28)**	8.0	8.0	10.0	**< 0.001**
0.25 and 0.75 quantiles	{7.0–9.0}	{6.0–9.0}	{8,8–10.0}	
IQR	2	3	1.2	

Bold *p*-values are significant, *IQR *interquartile range

Table 2 presents participants’ interest in dermatology on day 1 and day 28 based on gender (*n* = 48, corresponding to 71.6%, were females). There were no significant differences in the interest in dermatology on day 1 and day 28 between both groups.

**Table 2:** Comparison of participants’ interest in dermatology based on gender on day 1 and day 28 of the curriculum (*n* = 67)

	Total	Male	Female	*p*- value
**Participants** n (%)	67 (100)	19 (28.4)	48 (71.6)	
**Median Interest (day 1)**	7.0	7.0	7.0	**0.35**
0.25 and 0.75 quantiles	{6.0–8.0}	{5.0–8.0}	{5.0–8.0}	
IQR	2	3	2	
**Median Interest (day 28)**	8.0	8.0	8.0	**0.94**
0.25 and 0.75 quantiles	{7.0–9.0}	{7.0–9.0}	{7.0–9.0}	
IQR	2	2	2	

Bold *p*-values are significant, *IQR *interquartile range

### Positive aspects and suggestions to improve the curriculum

**Fig. 4:**
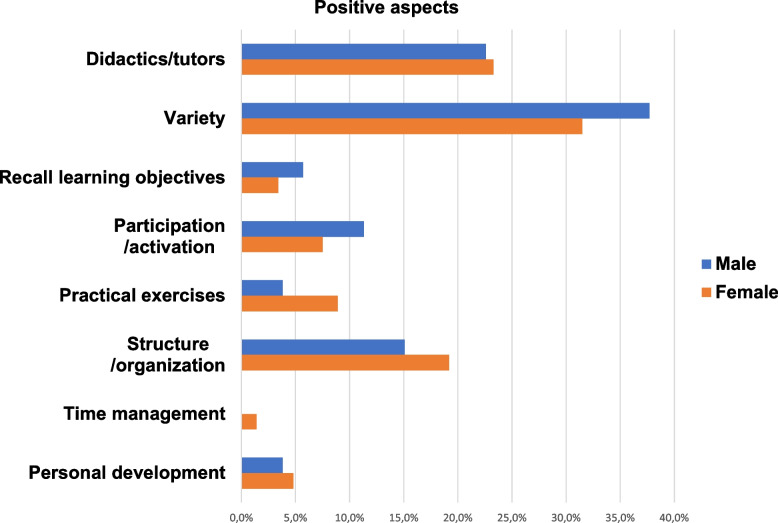
Distribution of individual responses by gender within the domain of positive aspects in %. In total 199 individual responses were given (*n* = 199), of these *n* = 53 from males and *n* = 146 from females

**Fig. 5:**
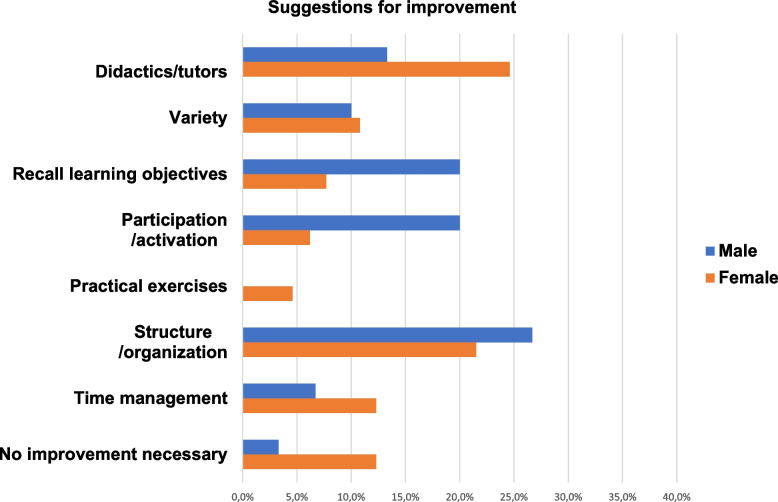
Distribution of individual responses by gender within the domain of suggestions for improvement in %. In total 95 individual responses were given (*n* = 95), of these *n* = 30 from males and *n* = 65 from females

Tables 3 and 4 show the aspects of the curriculum that participants rated as positive and suggestions for improvement. They were classified according to the above-mentioned categories. A presentation of the positive aspects and suggestions for improvement with regard to their distribution in the different categories is exhibited in an additional Fig. A1 (see Additional file 1). The distribution of the individual responses by gender in the different main categories are visualized in Fig. 4 for the positive aspects and in Fig. 5 for the suggestions for improvement.

**Table 3:** Categorization of the individual responses regarding positive aspects of the four-week curriculum in dermatology (*n* = 199)

Positive aspects of the curriculum by categories			
Category	Subcategory	n	%
Didactics/tutors	- Guidance, teaching, training, and feedback - Defined tutor - Flat hierarchies, tolerance	38 5 3	
		**46**	**23.1**
Variety	- Rotation in all functional areas - Various teaching materials (e.g., E-Learning, flashcards) - Varied ward work, different disease patterns - Extracurricular opportunities (e.g., research, congresses)	37 20 6 3	
		**66**	**33.2**
Recall learning objectives	- Checking learning objectives - Case presentation	6 2	
		**8**	**4.0**
Participation/ activation	- Independent and integrated work - Own and clear tasks with weekly goals	11 6	
		**17**	**8.5**
Practical exercises	- Practical tasks (e.g., biopsy, cryotherapy, assistance) - Own patient care and presentation to resident	8 7	
		**15**	**7.5**
Structure/ organization	- Clear structure and good organization - Good atmosphere and team	20 16	
		**36**	**18.1**
Time management	- Time for self-study - Time for patient care	1 1	
		**2**	**1.0**
Personal development	- Insight into dermatology - Personal knowledge consolidation	6 3	
		**9**	**4.5**
**Total count**		**199**

**Table 4:** Categorization of the individual responses regarding suggestions for improvement of the four-week curriculum in dermatology (*n* = 95)

Suggestions to improve the curriculum by categories			
Category	Subcategory	n	%
Didactics/tutors	- More guidance, teaching, training, and feedback - Defined tutor	19 1	
		**20**	**21.1**
Variety	- More rotation opportunities - Less traditional learning and more in-depth instruction	8 2	
		**10**	**10.5**
Recall learning objectives	- Meet query deadlines - More tests/queries/task control book - No case presentation	6 3 2	
		**11**	**11.6**
Participation/activation	- Introduction/explanation of tasks - More tasks/responsibilities	6 4	
		**10**	**10.5**
Practical exercises	- Practice of cutaneous injections - More own patient care and presentation to resident	1 2	
		**3**	**3.2**
Structure/organization	- Access to all IT platforms, phones, and work-related apps - Personal workspace - Fewer other undergraduates - Extend curriculum to 4 months	9 7 5 1	
		**22**	**23.2**
Time management	- No wasted time - Extra time for self-study - Flexible vacation planning	6 3 1	
		**10**	**10.5**
No improvement necessary		**9**	**9.5**
**Total count**		**95**

### Take-home messages

**Fig. 6:**
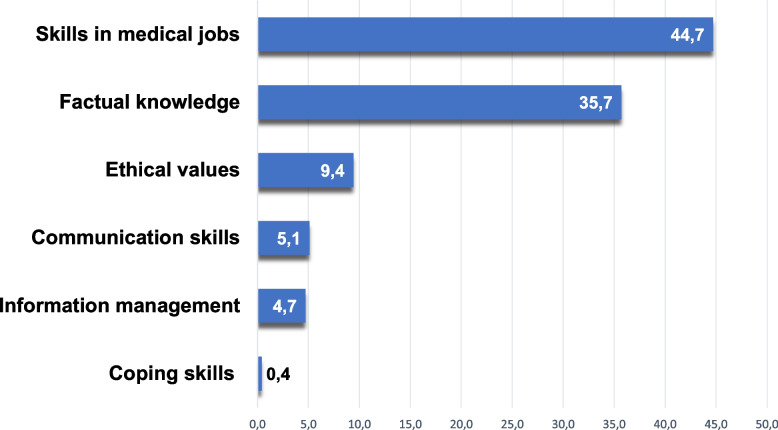
Distribution of take-home messages by categories in % (*n* = 255)

The distribution of take-home messages formulated by the participants regarding skills, knowledge, or other related abilities gained during the four-week curriculum is shown in Fig. 6. The category with the highest account of take-home messages was ‘skills in patient treatment’ accounting for 44.7%, followed by ‘factual knowledge’ with 35.7%. The categories with the lowest account of assigned take-home messages were ‘information management’ (4.7%) and ‘coping skills’ (0.4%).

## Discussion

In this single-center study, we introduced a newly created structured, four-week curriculum embedded into clinical routine of a tertiary dermatology university clinic and evaluated its impact on participants’ interest in dermatology and their reception of it. The mean year of birth of the 67 participants was 1995 and therefore represents an important interface to represent both the Generations Y and Z [9]. As reported previously, It can be challenging for healthcare institutions to meet the educational expectations of these generations [27, 28]. Hence, we were very interested in how the representatives of these new generations would receive our curriculum. With regard to gender distribution 71.6% of the participants were women, which corresponds to the demographic change in the medical profession in the Western world [29]. We therefore assume that the gender distribution in our study reflects the ongoing feminization in medicine that will hopefully result in more gender equality in medical leadership in the near future [30, 31].

We observed a significant increase in participants’ interest in dermatology over the course of the four-week curriculum, which aligns with our hypothesis that a well-structured curriculum including modern learning strategies may be attractive for students. When dividing the participants into five groups based on their interest in dermatology, our findings showed that those with initially lower levels of interest demonstrated an increase in interest during the curriculum, while those with higher initial interest levels maintained their high interest. This indicates that this curriculum might promote extrinsic motivation paralleled by enhanced intrinsic motivation, resulting in a very high interest for dermatology after 4 weeks. These results might be attributed to the integration of diversified, activating, and knowledge-testing teaching, patient observation, practical exercises, and e-learning tools. As a result, our curriculum appears to largely fulfill the key aspects of effective teaching recently described by Bernges et al. [17]. For instance, we incorporated various learning materials like flashcards [32], learning apps [33], or learning through practical exercises [34], whose effectiveness has been confirmed in other studies. It is the combination of such methods that is considered particularly effective [35, 36]. However, some authors suggest that it is not the medium itself that leads to learning success [37, 38], but rather the activation of knowledge acquisition through these activities [39]. Therefore, we extended activating teaching approaches, as recommended by Bernges et al., including case presentations, independent tasks with weekly objectives, and the practice and application of previously learned skills. We also conducted weekly, informal oral examinations to test learned knowledge, which were well appreciated by the participants. Thereby, we have focused on the didactic skill of the proficient tutor to ensure adequate student support to significantly improve the diagnostic skills in students as reported previously [13, 40].

Interestingly, the level of interest in dermatology was significantly higher in participants who were interested in doing a scientific work compared to their counterparts. This finding appears logical, as it can be assumed that participants with a high initial interest would try to deepen their interest in dermatology through conducting a scientific work. The fact that these participants were still highly interested in dermatology on day 28 confirms the assumption that students should be offered a wide range of opportunities at an early stage in order to motivate them and involve them in clinical practice as early as possible [41]. Publishing a scientific work can enhance one’s career and provide a solid entry into the professional world [42]. Furthermore, it was demonstrated that students participating in a scientific work developed a deeper understanding of critical scientific issues and data analysis [43]. Based on these considerations, the interest in scientific work could be a factor to identify students with a particularly strong interest in dermatology - which could be used as one of other selection criteria to offer students a training position in an institution.

Another aim of this study was to identify how to optimize future generations’ education in dermatology. Therefore, the positive aspects and suggestions to improve the curriculum where analyzed. The participants especially appreciated the ‘various teaching materials’ that were provided. While traditional lectures are less popular among students of the new generations [11], personalized learning units using modern technologies (e.g. smartphone applications, podcasts, video material) [44] as well as practical and creative activities are desired. In line with these findings, the ‘practical tasks’ applied in clinical practice were evaluated positively and the participants were very keen to work independently and take on ‘clear tasks’. The well-implemented teaching units, as well as the consistent feedback from the tutors and flat hierarchies within the department were also rated as positive. These findings are consistent with previous studies indicating that future generation students increasingly reject a hierarchical leadership style and instead wish a close mentor-student relationship that takes their needs seriously including regular feedback discussions [11, 12]. It is arguable whether such expectations and preferences should be met. Surely, they can be challenging to implement in clinical routine of most teaching institutions. At least, in our curriculum such expectations can be brought in line with clinical routine, resulting in a win-win situation both for the undergraduates and the institution. Moreover, many participating students highlighted the ‘good atmosphere’ in the team as an essential and positive factor. This finding is in accordance with other studies that emphasized the importance of teamwork for future generations [45, 46].

Undoubtedly, our curriculum is far from perfect. Various suggestions for improvement were made by the participants. Some of the criticism focused on similar areas as those highlighted in the list of positive aspects including flawless technology being of paramount importance or areas of structure/organization. For individuals of Generation Y and Z who grew up with the internet, smartphones, and social networks, this is an indispensable basic requirement [44, 47]. The access to modern technical devices and the use of effective software applications should therefore be standard for each teaching institution in the Western world. The critical use, medicolegal aspects, but also the huge potential of emerging fields in dermatology such as telemedicine, artificial intelligence, and information through social media should be incorported into the medical training, as they are becoming indispensable areas of medicine in general. At the same time confidentiality issues have to be considered. In our institution, undergraduates are informed on day 1 that taking photographs of patients using private devices is strictly forbidden as recommended by ethical standards [48, 49]. However, dysfunctional electronic equipment might entice hospital staff to using their private devices- another reason to ensure smoothly functional work units [50]. System failures, data security concerns, limited financial resources, as well as a lack of expertise and reluctance to innovate established practices, pose significant questions for the entire medical system [51–53]. Alongside these developments, all involved parties are facing new responsibilities and challenges. The new generations of ‘digital natives’, in particular, represent a great potential to address these issues, which should be recognized and harnessed by medical teachers.

In terms of the category ‘time management’, extra time for self-study and more flexibility were suggested. These results are in line with the wishes of the Generation Y and Z for more flexible working hours, in terms of place and time, as this contributes to a better work-life balance [12]. According to Raines et al., younger colleagues are exposed to criticism from older generations, who often misinterpret these demands as low work ethic or lack of motivation [47]. Future generations also pay less attention to exact compliance with working hours but focus on effective work in the shortest possible time, [54] which can come across peculiar.

Key issues raised in the category ‘didactics and tutors’ included achieving more variety through ‘more specific teaching units’ or better didactics through ‘more guidance’. These inputs align with the previously discussed desires for high-quality supervision by senior doctors including feedback, practical experiences, and workplace learning.

While studies from of the last decade show differences between the two genders in terms of career and education aspirations [55–57], the question is whether these are becoming less distinct nowadays. Our findings may assume difference such as male participants greater wish for active participation and review of learning objectives, while females requested for more teaching/instruction and proper time management. But due to the small number of participants in the study, it is difficult to make a statement regarding its significance and causality, also due to the growing proportion of women in the medical field [29, 31, 55]. Thus, further studies are necessary to address the role of gender in the context of dermatology training in medical students.

Formulating personal take-home messages has the potential to establish individual key moments throughout the course of medical studies. While learning objectives can be formulated in curricula, the measure of learning success becomes clearer when students consider the information, they have acquired to be important and formulate them as personal take-home messages. These could also have an influence on the choice of specialization during their further careers. A clear strength of the curriculum was the imparting of skills relevant for non-dermatologists treating dermatology patients. Student responses affirmed successful learning, and some displayed remarkable interest in dermatology. The incorporation of soft skills, such as addressing ethical values or learning communication skills, could only be partially conveyed to the participants. Studies indicate that ethical issues such as environmental awareness, social justice [11, 44], and a healthy work environment [44] will become increasingly important for the future Generations Y and Z, highlighting the need to integrate such topics within the curriculum.

To the best of our knowledge, the curriculum presented is the first one published for advanced medical students. Particular importance was placed on the students’ perceptions and suggestions for improvement. The authors of previous studies highlight similar focal points, which largely align with the content and aims of our curriculum [58–60]. The importance of conveying the skills for history taking, describing skin lesions, performing diagnostics including reviewing differential diagnoses and providing appropriate treatment was also emphasized by the other authors [58, 59]. Moreover, they underlined the importance of active knowledge retrieval through assessments and the use of appropriate evaluation methods [58, 60]. Additionally, they highlighted the value of feedback discussions with a mentor [58], collaborative review and discourse on individual patient cases [58], and participation in practical clinical activities under mentor guidance [58, 59]. Irrespective of the specialty, previous studies demonstrated the necessity of a core curriculum to establish uniform minimum standards of knowledge and skills for medical students, as well as to make their individual progress visible for themselves [17, 61]. Based on our results, we may state that our curriculum includes all these aspects, at least to some extent.

To achieve these goals, an analysis is needed that combines subjective criteria (e.g., self-assessments), objective criteria (e.g., knowledge tests), and student and tutor perceptions and suggestions for improvement. Since our study focuses on the subjective feedback and individual improvement suggestions of students, it might not capture the complete picture. Objective measures of knowledge acquisition or performance would have provided a more holistic picture. Adding a control group would have consolidated our results, and the use of a psychometric tool would have ensured the accuracy of the data. Therefore, these elements should be integral parts of the study design in future research. The applicability of individual learning modules should be confirmed in further studies so that they can be easily integrated into other countries and curricula. However, student perceptions and feedback are underrepresented in research [3, 20], making our study an important basis in the professionalization of medical education in dermatology.

## Strength and limitations

The strengths of this study include its focus on the perception of the curriculum from the undergraduate students’ perspective and the investigation of a modern curriculum that is well integrated into our clinical practice. The purely subjective survey without objective criteria represents a limitation of this study. The form of data collection did not allow any reliability or validity to be tested*.* In addition, only the students were surveyed, while the assessment of the tutors was not a standardized part of our design. However, according to verbal feedback, the tutors greatly appreciated their teaching activities in the curriculum. Another limitation is the single-center design and the lack of a control group, making it challenging to differentiate whether all the observed changes in interest or knowledge acquisition are solely attributable to the curriculum. Finally, the results and the presented curriculum itself might not be generalizable to other countries due to differences in the structure of the medical studies, organization and duration of student rotations into dermatology clinics.

## Conclusion

The study findings suggest that the presented four-week curriculum significantly enhanced students’ interest in dermatology and provided them with important skills and knowledge for their future medical work. We conclude that this modular curriculum is didactically effective to both motivate and satisfy students of the Generation Y and Z during clinical routine. Therefore, this curriculum could serve as a model for other dermatology teaching institutions but, depending on the medical education system, it might need country-specific and institution-adapted modifications.

### Supplementary Information

**Additional file 1.**

**Additional file 2.**

## Data Availability

The datasets generated and/or analyzed during the current study are not publicly available due to ethical restrictions but are available from the corresponding author on reasonable request.
