# Supplementary material for: Dermatology teaching for undergraduate medical students in clinical routine – a structured four-week curriculum
Source: BMC Med Educ. 2024 Feb 6;24:116. doi: 10.1186/s12909-023-04921-x (PMC10848555; doi:10.1186/s12909-023-04921-x)
Supplement: Supplementary file 1 — Additional file 1. [file 12909_2023_4921_MOESM1_ESM.docx]

**Additional File 1**

**Table A1.** Comparison of the participants` median interest in dermatology at different points in time (n=67).

|  | **Day 1** | **Day 7** | **Day 14** | **Day 21** | **Day 28** |
| --- | --- | --- | --- | --- | --- |
| **Median Interest (n=67)**  0.25 and 0.75 quantiles  IQR | 7.0  {6.0-8.0}  2 | 7.0  {6.0-8.0}  2 | 7.0  {6.0-9.0} 3 | 8.0  {6.0-9.0}  3 | 8.0  {7.0-9.0}  2 |
| Post-hoc-testing  p-values in relation to day 1 |  | 1.00 | 0.59 | **0.001** | **<0.001** |
| Post-hoc-testing  p-values compare current measurement time point with previous measurement time point |  | 1.00 | 1.00 | 0.33 | 1.00 |

*Bold p-values are significant, IQR = interquartile range*

**Figure A1a and b**. Distribution of the positive aspects (a) and suggestions to improve (b) the curriculum according to categories.

Figure A1a: Positive aspects of the curriculum by categories

Figure A1b: Suggestions to improve the curriculum by categories

**Table A2.** Distribution of individual responses by gender within the domains of positive aspects and suggestions for improvement. 'n' represents the number of individual responses provided.

|  | **Total** | | **Male** | | | **Female** | |
| --- | --- | --- | --- | --- | --- | --- | --- |
| **Positive aspects** | **n**  (199) | **%** | **n**  (53) | **%** | | **n**  (146) | **%** |
|  |  | | | | | | |
| Didactics/ tutors | 46 | 23.1 | 12 | | 22.6 | 34 | 23.3 |
| Variety | 66 | 33.2 | 20 | | 37.7 | 46 | 31.5 |
| Recall learning objectives | 8 | 4.0 | 3 | | 5.7 | 5 | 3.4 |
| Participation/ activation | 17 | 8.5 | 6 | | 11.3 | 11 | 7.5 |
| Practical exercises | 15 | 7.5 | 2 | | 3.8 | 13 | 8.9 |
| Structure/ organization | 36 | 18.1 | 8 | | 15.1 | 28 | 19.2 |
| Time management | 2 | 1.0 | 0 | | 0.0 | 2 | 1.4 |
| Personal development | 9 | 4.5 | 2 | | 3.8 | 7 | 4.8 |
|  |  | | | | | | |
| **Suggestions for improvement** | **n**  (95) | **%** | **n**  (30) | | **%** | **n**  (65) | **%** |
|  |  | | | | | | |
| Didactics/ tutors | 20 | 21.1 | 4 | | 13.3 | 16 | 24.6 |
| Variety | 10 | 10.5 | 3 | | 10.0 | 7 | 10.5 |
| Recall learning objectives | 11 | 11.6 | 6 | | 20.0 | 5 | 7.7 |
| Participation/ activation | 10 | 10.5 | 6 | | 20.0 | 4 | 6.2 |
| Practical exercises | 3 | 3.2 | 0 | | 0.0 | 3 | 4.6 |
| Structure/ organization | 22 | 23.2 | 8 | | 26.7 | 14 | 21.5 |
| Time management | 10 | 10.5 | 2 | | 6.7 | 8 | 12.3 |
| No improvement necessary | 9 | 9.5 | 1 | | 3.3 | 8 | 12.3 |
