# Supplementary material for: Dermatology teaching for undergraduate medical students in clinical routine – a structured four-week curriculum
Source: BMC Med Educ. 2024 Feb 6;24:116. doi: 10.1186/s12909-023-04921-x (PMC10848555; doi:10.1186/s12909-023-04921-x)
Supplement: Supplementary file 2 — Additional file 2. [file 12909_2023_4921_MOESM2_ESM.docx]

**Unterassistenten-Curriculum Dermatologie**

*Ärztliches Kader:*

Prof. Dr. Dr. Alexander Navarini

Prof. Dr. Karin Hartmann, LA Allergologie

Dr. Michael Kunz, Kaderarzt

PD Dr. Beda Mühleisen, Kaderarzt

PD Dr. Simon Müller, Kaderarzt

*Unterassistentin/Unterassistent:*

*Ansprechperson Administration/Planung:*

*Ansprechperson AA:*

*Ansprechperson OA:*

| ***Wochenziele/Inhalt*** | ***Lerninhalte*** | ***Findet statt*** | ***Bemerkungen*** |  |
| --- | --- | --- | --- | --- |
| 1. Woche | **Effloreszenzenlehre** | Am:  wird abgefragt von | Informationsquelle:  Handout zur Effloreszenzenlehre  Nast et al., BJD 2017  Häufiges Üben in Poliklinik und Abt. | **Täglich 5 Lernkarten bearbeiten**  **«Fitzpatricks Dermatology Flash Cards»** |
| 2. Woche | **Anamnese, Klinik, Diagnostik und Therapiekonzepte von häufigen Dermatosen**   - Akne - Alopecia areata - Atopische Dermatitis - Herpes simplex und Herpes zoster - Kontaktdermatitis (allergisch/irritativ-toxisch) - Non-melanoma skin cancers (Basalzell- und Plattenepithel-CAs) - Psoriasis - Tinea/Onychomykosen - Urticaria - Verrucae vulgares | Am:  wird abgefragt von | Informationsquelle:  «Dermatology Essentials»  *Jean L. Bolognia*  (elektronisches PDF von L. Gehring) |  |
| 3. Woche  Poliklinik und/oder Abteilung | **Anwendung von theoretischen Lerninhalten und Übung in Gesprächsführung, Blickdiagnostik, dermatologischen Tests** (z.B. Diaskopie, Dermographismus, Direktpräparat, Psoriasis-Zeichen, Hobelspan-Phänomen, Apple-Jelly-Phänomen, Wood-Licht etc.) |  |  |  |
| 4. Woche  Vorstellung  **«Mein lehrreichster Fall»** | (PowerPoint-Präsentation, die ich für andere UAs halte, 15 Min.)  **Synthese von Theorie, Praxis, Erfahrung und Vortrags-Skills**  **Mein Thema:** | Am:  Wo: Online  unter Moderation von: |  | **Täglich 5 Lernkarten**  **«Fitzpatricks Dermatology**  **Flash Cards»** |

**Mein Interesse an der Dermatologie auf einer Skala von 0 bis 10** (0 = kein Interesse / 10 = es kommt keine andere Disziplin in Frage)

Am 1. Tag: Nach 1 Woche: Nach 2 Wochen: Nach 3 Wochen: Nach 4 Wochen:

**Ich habe Interesse, während meiner UA-Zeit ein kleines wissenschaftliches Projekt zu machen (kann auch nach Ende der UA-Zeit finalisiert werden):**

Nein Ja Falls ja, bitte in den ersten 3 Tagen Kontaktaufnahme

**Meine wichtigsten 3 Take Home Messages am Ende der UA-Zeit in dieser Abteilung sind:**

**1.**

**2.**

**3.**

**Was könnte man an diesem UA-Curriculum noch verbessern?**

**Was hat mir besonders gut an diesem UA-Curriculum gefallen?**

Bitte dieses Formular am Ende der UA-Zeit im Sekretariat abgeben.

**My interest in dermatology on a scale from 0 to 10** (0 = no interest / 10 = no other discipline comes into question)

On day one: After one week: After two weeks: After three weeks: After four weeks:

**I am interested in doing a small scientific project during my internship time (can also be finalized after the end of my internship time):**

No Yes If yes, please contact ___ within the first 3 days.

**My top 3 take home messages at the end of the internship time in this department are:**

**1.**

**2.**

**3.**

**What could be improved in this curriculum?**

**What did I particularly like about this curriculum?**

Please return this form to the secretary's office at the end of the internship.
